# Health care professionals’ experiences of dealing with cancer cachexia

**DOI:** 10.1007/s10147-023-02300-6

**Published:** 2023-02-23

**Authors:** Jodie Ellis, Michelle Petersen, Sungwon Chang, Gemma Ingham, Peter Martin, Nicola Morgan, Vanessa Vaughan, Linda Brown, David C. Currow, Valentina Razmovski-Naumovski

**Affiliations:** 1 Calvary Health Care Kogarah, Kogarah, NSW 2217 Australia; 2 IMPACCT-Improving Palliative, Aged and Chronic Care Through Clinical Research and Translation, Faculty of Health, University of Technology, Sydney, NSW 2007 Australia; 3 Wollongong Hospital, Wollongong, NSW 2500 Australia; 4 The Prince of Wales Hospital, Sydney, NSW 2031 Australia; 5 School of Medicine, Faculty of Health, Deakin University, Geelong, VIC 3216 Australia; 6 Gold Coast Hospital and Health Service, Southport, QLD 4215 Australia; 7 Faculty of Health Sciences and Medicine, Bond University, Gold Coast, QLD 4226 Australia; 8 The Faculty of Science, Medicine and Health, University of Wollongong, Wollongong, NSW 2522 Australia; 9 South West Sydney Clinical Campuses, Faculty of Medicine and Health, University of New South Wales (UNSW), Medical Education and Research Precinct, Level 2, Clinical Building, Liverpool Hospital, Cnr Elizabeth and Goulburn Sts, Liverpool, NSW 2170 Australia; 10 Ingham Institute of Applied Medical Research, Liverpool, NSW 2170 Australia

**Keywords:** Cachexia, Cancer, Experiences, Health care professional, Management, Survey

## Abstract

**Background:**

Cancer cachexia (CC) is a debilitating syndrome severely impacting patients’ quality of life and survivorship. We aimed to investigate the health care professionals’ (HCPs’) experiences of dealing with CC.

**Methods:**

Survey questions entailed definitions and guidelines, importance of CC management, clinician confidence and involvement, screening and assessment, interventions, psychosocial and food aspects. The online survey was disseminated through Australian and New Zealand palliative care, oncology, allied health and nursing organisations. Frequencies were reported using descriptive statistics accounting for response rates. Associations were examined between variables using Fisher’s exact and Pearson’s chi-square tests.

**Results:**

Over 90% of the respondents (*n* = 192) were medical doctors or nurses. Over 85% of the respondents were not aware of any guidelines, with 83% considering ≥ 10% weight loss from baseline indicative of CC. CC management was considered important by 77% of HCPs, and 55% indicated that it was part of their clinical role to assess and treat CC. In contrast, 56% of respondents were not confident about managing CC, and 93% believed formal training in CC would benefit their clinical practice. Although formal screening tools were generally not used (79%), 75% of respondents asked patients about specific symptoms. Antiemetics (80%) and nutritional counselling (86%) were most prescribed or recommended interventions, respectively.

**Conclusion:**

This study underlines the deficiencies in knowledge and training of CC which has implications for patients’ function, well-being and survival. HCP training and a structured approach to CC management is advocated for optimal and continued patient care.

**Supplementary Information:**

The online version contains supplementary material available at 10.1007/s10147-023-02300-6.

## Introduction

Cancer cachexia (CC) is a multifactorial metabolic syndrome encompassing appetite and weight loss and severe muscle wasting [1, 2]. Cachexia is estimated in 80% of people with advanced cancer [3–5], and is implicated in around 20% of cancer deaths [6]. CC is associated with the reduced tolerance of ongoing cancer treatments, increased toxicity and treatment delays and is an independent predictor of survival [7–9]. Reduced physical, social and psychological functioning [10] affects the quality of life of the patient and causes distress for carers who see this as a harbinger of death [11].

While it is a prevalent and devastating syndrome [12], cachexia assessment and management remains an unmet medical need for many people with cancer [2, 12–14]. Previously, three global surveys undertaken across 14 countries investigated goals of treatment practices and highlighted the need for the increased awareness and management of CC among health care professionals (HCPs) [13]. Semi-structured interviews undertaken in a dedicated cachexia clinic concurred that improved knowledge would increase staff confidence to broach CC with patients and their carers [15]. Another focus group/semi-structured study added that culture and available resources underpinned a holistic model of care approach [16]. Although this research has outlined the need for more attention in tackling CC, little is known about the current understanding and work practices of HCPs regarding CC management. Thus, this study investigated the HCPs’ experiences of dealing with CC. Furthermore, we examined whether previous survey findings extended to Australia and New Zealand.

## Methods

Forty-six multi-choice and 18 other/open questions were formulated to reflect current CC knowledge [13, 15, 16] under the headings: definitions and guidelines; importance of CC management; HCP confidence and involvement; screening and assessment tools; pharmaceutical and nonpharmaceutical interventions; psychosocial and food aspects.

As the first local initiative, there was no external validation of the questions. The survey was constructed in REDCap (Vanderbilt University, U.S.A.) and tested prior to dissemination. The survey link was sent to oncology, palliative care and allied health organisations (*n* = 9) which was then distributed to their respective memberships. The survey was open between 29th September 2018 and 26th July 2019. This study was performed in line with the principles of the Declaration of Helsinki. Approval was obtained from the Faculty of Health Low Risk Ethics Panel (Reference number ETH18-2870), University of Technology Sydney, Australia. Informed consent was obtained from all individual participants included in the study.

Data were analysed using SPSS Statistics software (V19; Armonk, NY: IBM Corp). Frequencies were described, and the results were reported using descriptive statistics accounting for response rates. Fisher’s exact and Pearson chi-square tests examined the association between two or more variables, respectively. *P* < 0.05 showed an association between the variables. Responses of other/open questions were tabulated and summarised (Supplementary 1).

## Results

### Survey participation and respondent’s demographics

**Fig. 1:**
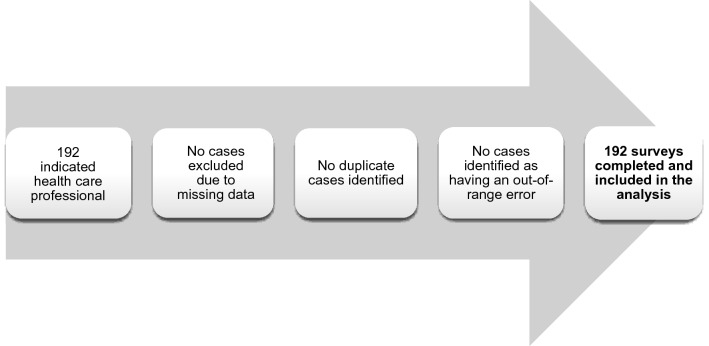
Data process to reach full analysis set

A flow diagram for the data process is shown in Fig. 1. In this study, 192 respondents indicated that they were HCPs and were included in the analysis. Most respondents lived in Australia, worked in palliative care and were medical doctors or nurses (Table 1). The results were summarised into three main themes: knowledge, clinical practice and clinical management in CC.

**Table 1:** Demographics of clinician respondents included in the health care professional experiences of dealing with cancer cachexia study in Australia and New Zealand (*n* = number of responders)

Demographics of responders (*n* = number of responders)	Responses (percentage %)
**Country of work** (***n*** **= 192**)	
Australia	172 (89.6)
New Zealand	20 (10.5)
**Occupation** (***n*** **= 192**)	
Medical doctor	86 (44.8)
Area of practice (*n* = 86)^a^	
Palliative care	79 (91.9)
Oncology	5 (5.8)
Other	2 (2.3)
Highest level of training (*n* = 84)^b^	
Specialist	70 (83.3)
Registrar	11 (13.1)
Other	3 (3.6)
Nursing	87 (45.3)
Dietitian	9 (4.7)
Physiotherapist	5 (2.6)
Occupational therapist	2 (1.0)
Psychologist/social worker	1 (0.5)
Other	2 (1.0)
**Current work location** (***n*** **= 192**)	
Metropolitan city/centre	127 (66.1)
Regional city/centre	49 (25.5)
Rural town/centre	23 (12.0)
Remote area	2 (1.0)
**Current workplace** (***n*** **= 192**)	
Palliative care unit	62 (32.3)
Acute hospital (Palliative care)	56 (29.2)
Acute hospital (Oncology)	35 (18.2)
Community/outpatient (Palliative care)	60 (31.3)
Community/outpatient (Oncology)	22 (11.5)
Other	19 (9.9)
**Health system **(***n* = 192**)	
Public health system only^c^	146 (76.0)
Private health system only^d^	16 (8.3)
Mixed public/private	30 (15.6)

^a^Area of clinical practice in which the medical doctor primarily focusses their career

^b^After graduation, training includes Internship and Residency. This is followed by enrolment into a specialist training programme as a Registrar. This eventually leads to fellowship qualification and recognition as a fully qualified Specialist Medical Doctor

^c^In Australia, public hospitals are owned and managed by state and territory governments, with Medicare providing access to free treatment and accommodation in a public hospital

^d^Private hospitals are owned and operated by the private sector but licensed and regulated by governments

### Knowledge

#### Awareness of definitions and guidelines for cancer cachexia

Thirty-five percent of respondents reported awareness of the consensus definition of CC (*n* = 176) [2] however, 83% incorrectly reported that weight loss of  ≥ 10% corresponded to CC (*n* = 174). HCPs were aware of the European Society for Clinical Nutrition and Metabolism (ESPEN) guidelines (*n* = 165) [17] (Table 2). Complementing this finding, 80% and 91% of respondents wanted a better understanding of the physiological processes and current CC guidelines, respectively.

**Table 2:** Knowledge: Awareness of definitions and guidelines for cancer cachexia, confidence in managing the syndrome and comfort in discussing psychosocial aspects of it (*n* = number of responders)

Awareness of:	Yes (%)	No (%)
2011 consensus definition of cancer cachexia [2]^a^ (*n* = 176)	62 (35.2)	114 (64.8)
ESPEN^b^ guidelines on nutrition in cancer patients [17] (*n* = 165)	24 (14.5)	141 (85.5)
Other guidelines (including local guidelines) (*n* = 166)	22 (13.3)	144 (86.7)

	< 10%	10%	20%	30%	> 30%
At what percentage of weight loss from baseline do you consider a patient to have cancer cachexia? (*n* = 174)	30 (17.2)	68 (39.1)	42 (24.1)	14 (8.0)	20 (11.5)

How confident do you feel in your ability to manage people with advanced cancer who have cachexia? (*n* = 135)	Extremely confident *n* (%)	Somewhat confident *n* (%)	Neutral *n* (%)	Somewhat unconfident *n* (%)	Extremely unconfident *n* (%)
	5 (3.7)	55 (40.7)	26 (19.3)	37 (27.4)	12 (8.9)

How comfortable are you with discussing the psychosocial impact of cancer cachexia with: (*n* = 133)	Extremely comfortable *n* (%)	Somewhat comfortable *n* (%)	Neutral *n* (%)	Somewhat uncomfortable *n* (%)	Extremely uncomfortable *n* (%)
Patients	39 (29.3)	55 (41.4)	27 (20.3)	9 (6.8)	3 (2.3)
Carers	41 (30.8)	63 (47.4)	20 (15)	6 (4.5)	3 (2.3)

^a^More than 5% weight loss over 6 months

^b^*ESPEN* European Society for Clinical Nutrition and Metabolism

#### Clinician confidence and training in cancer cachexia

Fifty-six percent of respondents were neutral or lacked confidence in managing people with CC (*n* = 135) (Table 2). Ninety-two percent (*n* = 131) indicated that a central place for CC information would aid clinicians’ confidence in managing and discussing CC. Over 70% of the HCPs were comfortable discussing the psychosocial impact of CC with patients and their carers (Table 2). Only 16% reported receiving any training in CC, and 93% indicated that formal training or education would benefit their clinical practice. Ninety-seven percent (*n* = 147) agreed that further clinical research in CC and its impact on patients was required.

### Clinical practice

#### Clinician involvement: use of a multidisciplinary team

Eighty-eight percent (*n* = 135) of respondents reported that multidisciplinary team meetings were part of their current practice, with the following personnel involved (*n* = 119): medical doctors (92%), nursing (98%), dietitian (48%), physiotherapist (63%), occupational therapist (64%), psychologist/social worker (78%), counsellor (29%), speech pathologist (24%) and other (24%). Assessment (56%), treatment (59%) and monitoring (51%) were discussed (*n* = 192). Table 3 shows the frequency of these HCPs’ involvement with patients, with medical doctors and nurses involved in “regular review”, whilst other personnel were involved “as needed”.

**Table 3:** Clinical practice: Personnel involvement, screening and ongoing assessment for people at risk of cancer cachexia (CC) (*n* = number of responders)

Personnel				
	When are different disciplines engaged? (*n* = 119)			
Personnel involved in multidisciplinary team meetings (%)	Initial only *n* (%)	Regular review *n* (%)	As needed *n* (%)	Not applicable *n* (%)
Medical doctor (92)	9 (7.6)	70 (58.8)	35 (29.4)	5 (4.2)
Nursing (98)	3 (2.5)	86 (72.3)	29 (24.4)	1 (0.8)
Dietitian (48)	3 (2.5)	24 (20.2)	66 (55.5)	26 (21.8)
Physiotherapist (63)	2 (1.7)	28 (23.5)	70 (58.8)	19 (16.0)
Occupational therapist (64)	2 (1.7)	20 (16.8)	79 (66.4)	18 (15.1)
Psychologist/Social Worker (78)	3 (2.5)	37 (31.1)	72 (60.5)	7 (5.9)
Counsellor (29)	1 (0.8)	9 (7.6)	60 (50.4)	49 (41.2)
Speech pathologist (24)	3 (2.5)	7 (5.9)	73 (61.3)	36 (30.3)
Other (24) (*n* = 67)	1 (1.5)	5 (7.5)	17 (25.4)	44 (65.7)

Current screening practice		Yes *n* (%)	No *n* (%)
Is it part of your current practice to assess and treat CC? (*n* = 143)		79 (55.2)	64 (44.8)
Do you use a formal nutrition screening tool for CC? (*n* = 79)		17 (21.5)	62 (78.5)
If ‘yes’ to using a formal nutritional tool (*n* = 17)	Mini Nutrition Assessment Short Form (MNA SF)	1 (5.9)	
	Malnutrition Universal Screening Tool (MUST)	2 (11.8)	
	Malnutrition Screening Tool (MST)	13 (76.5)	
	Nutrition Risk Screening (NRS—2002)	3 (17.6)	
	Other	3 (17.6)	
Do you screen for specific symptoms related to CC? (*n* = 79)		59 (74.7)	20 (25.3)
If ‘yes’ to screening for specific symptoms (*n* = 59)	Nausea/vomiting	58 (98.3)	
	Poor appetite	59 (100)	
	Early satiety	48 (81.4)	
	Anxiety (weight loss related)	50 (84.7)	
	Diarrhoea	49 (83.1)	
	Constipation	56 (94.9)	
	Candidiasis	51 (86.4)	
	Dysphagia	53 (89.8)	
	Odynophagia	42 (71.2)	
	Fatigue (with oral intake)	54 (91.5)	
	Other	10 (16.9)	
Do you reassess these specific symptoms at each review? (*n* = 59)		44 (74.6)	15 (25.4)
Do you use pathology tests for screening for CC? (*n* = 79)		34 (43.0)	45 (57.0)
If ‘yes’ to using pathology tests for screening (*n* = 34)	Albumin/Pre-Albumin	34 (100)	
	C-reactive protein/erythrocyte sedimentation rate (CRP/ESR)	20 (58.8)	
	Haemoglobin (Hb)	25 (73.5)	
	Blood sugar level (BSL)	20 (58.8)	
	Calcium	23 (67.6)	
	Testosterone	3 (8.8)	
	Vitamin D	9 (26.5)	
	Other	2 (5.9)	
Do you use biometric measures for screening for CC? (*n* = 79)		26 (32.9)	53 (67.1)
If ‘yes’ to using biometric measures for screening (*n* = 26)	Weight	26 (100)	
	Body Mass Index (BMI)	19 (73.1)	
	Mid arm circumference	3 (11.5)	
	Hand grip strength	4 (15.4)	
	Dual energy X-ray absorptiometry (DEXA) scan	1 (3.8)	
	Other	6 (23.1)	

Ongoing assessment				
Use of assessment tools over time (*n* = 63)	Initial only *n* (%)	Regular review *n* (%)	As needed *n* (%)	Not applicable *n* (%)
Patient Generated Subjective Global Assessment (PG-SGA)	5 (7.9)	9 (14.3)	3 (4.8)	46 (73.0)
Subjective Global Assessment (SGA)	1 (1.6)	9 (14.3)	5 (7.9)	48 (76.2)
Mini Nutrition Assessment (MNA)	2 (3.2)	5 (7.9)	6 (9.5)	50 (79.4)
Functional Assessment in Anorexia/Cachexia Treatment (FAACT)	0	5 (7.9)	4 (6.3)	54 (85.7)
Specific symptom questions	7 (11.1)	41 (65.1)	10 (15.9)	5 (7.9)
Pathology tests	4 (6.3)	17 (27.0)	28 (44.4)	14 (22.2)
Biometric tests	2 (3.2)	12 (19.0)	15 (23.8)	34 (54.0)
Other	2 (3.2)	4 (6.3)	3 (4.8)	54 (85.7)

Forty-two percent (*n* = 119) reported that specific personnel were missing from the multidisciplinary team (*n* = 49): medical doctors (10%), nursing (4%), dietitian (57%), physiotherapist (41%), occupational therapist (29%), psychologist/social worker (16%), counsellor (37%), speech pathologist (61%) and other (16%).

#### Screening for cancer cachexia

Fifty-five percent of respondents assessed and treated CC in their practice (*n* = 143), with only 17 (22%, *n* = 79) using formal nutrition screening tools (Table 3). The Malnutrition Screening Tool (MST) was used by 77% (of *n* = 17). Seventy-five percent (*n* = 79) screened for specific symptoms related to CC, with 75% reassessing the symptoms at each review (*n* = 59). All respondents screened for poor appetite.

Pathology tests for CC screening (*n* = 79) was used by 43% of respondents. Most frequently prescribed tests were albumin/pre-albumin, haemoglobin, calcium, C-reactive protein/erythrocyte sedimentation rate (CRP/ESR) and blood sugar level. Thirty-three percent reported using biometric tests (*n* = 79), with weight (100%) and body mass index (BMI) (73.1%) most measured.

#### Assessment tools used in cancer cachexia

Forty-five percent of respondents monitored CC beyond initial screening (*n* = 139). Many of these assessment tools (Table 3) were rated as “not applicable” (> 70%). Standard practice assessments such symptom monitoring was “ongoing” (65%), whilst pathology (44%) and biometric assessments were “as needed” (24%).

### Clinical management

#### Importance of cancer cachexia management

Seventy-seven percent of respondents indicated that CC management was an important part of their practice (*n* = 149), with two thirds of this sub-group noting that it was important when compared to other symptoms (Table 4). Carers (40%) were more likely to initiate discussions regarding CC management.

**Table 4:** Clinical management: Importance of cancer cachexia management to health care professionals and prescription or recommended treatment options for cancer cachexia

Importance of cancer cachexia management to health care professionals	Extremely important *n* (%)	Somewhat important *n* (%)	Neutral *n* (%)	Somewhat unimportant *n* (%)	Extremely unimportant *n* (%)
Is cancer cachexia management an important part of your clinical practice? (*n* = 149)	46 (30.9)	69 (46.3)	23 (15.4)	8 (5.4)	3 (2.0)
How important is cancer cachexia symptom management compared to other symptoms (e.g. pain, nausea etc.)? (*n* = 149)	41 (27.5)	58 (38.9)	28 (18.8)	20 (13.4)	2 (1.3)

	You *n* (%)	Patient *n* (%)	Caregiver *n* (%)	GP^a^ *n* (%)	Other medical/allied health *n* (%)
Who is more likely to initiate discussion/management of cancer cachexia? (*n* = 149)	48 (32.2)	20 (13.4)	60 (40.3)	3 (2.0)	18 (12.1)

Prescription or recommended treatment options for cancer cachexia (*n* = 140*)	Extremely likely *n* (%)	Somewhat likely *n* (%)	Neutral *n* (%)	Somewhat unlikely *n* (%)	Extremely unlikely *n* (%)
How likely are you to prescribe (or recommend to a doctor to prescribe) the following for the management of cancer cachexia?					
Antiemetics	59 (42.1)	53 (37.9)	16 (11.4)	4 (2.9)	8 (5.7)
Steroids	22 (15.7)	64 (45.7)	20 (14.3)	18 (12.9)	16 (11.4)
Megestrol acetate	1 (0.7)	12 (8.6)	38 (27.1)	35 (25.0)	54 (38.6)
Androgens	1 (0.7)	1 (0.7)	33 (23.6)	28 (20.0)	77 (55.0)
Prokinetics	31 (22.1)	47 (33.6)	33 (23.6)	3 (2.1)	26 (18.6)
Thalidomide	0	2 (1.4)	26 (18.6)	20 (14.3)	92 (65.7)
Cannabinoids	2 (1.4)	22 (15.7)	35 (25.0)	32 (22.9)	49 (35.0)
Anamorelin	0	5 (3.6)	34 (24.3)	25 (17.9)	76 (54.3)
Others (*n* = 33)					
How likely are you to recommend the following for managing cancer cachexia?					
Exercise	35 (25)	50 (35.7)	27 (19.3)	15 (10.7)	13 (9.3)
Nutritional counselling	80 (57.1)	41 (29.3)	9 (6.4)	5 (3.6)	5 (3.6)
Psychological support (*n* = 139)*	42 (30.2)	71 (51.1)	16 (11.5)	7 (5.0)	3 (2.2)
Mouth care	79 (56.4)	43 (30.7)	11 (7.9)	5 (3.6)	2 (1.4)
Complementary/alternative medicines	9 (6.4)	21 (15)	46 (32.9)	36 (25.7)	28 (20)
Other (*n* = 15)					

*139 respondents answered, not 140 for psychological support

^a^*GP *General practitioner/family doctor

#### Pharmaceutical and nonpharmaceutical interventions

Antiemetics, prokinetic agents and steroids were the most prescribed medications, with megestrol acetate less popular (Table 4). Agents such as androgens, anamorelin, cannabinoids and thalidomide were less frequently prescribed. Over 80% of respondents recommended the nonpharmaceutical management of CC including nutritional counselling, psychological support and mouth care.

#### Food aspects

Restrictive eating practices (e.g. eliminating refined sugar/gluten free diet/vegan diet/alkaline diet etc.) and cultural attitudes towards food were likely to be asked by 54% and 59% of the respondents, respectively (*n* = 133).

### Statistical analysis

Fisher’s exact test results (Table 5) showed an association with the 2011 definition and pathology tests (*p* = 0.01); ESPEN Guidelines with formal nutritional tool (*p* = 0.04); current practice to assess and treat CC and awareness of the ESPEN Guidelines or other/local guidelines (both *p* = 0.00).

**Table 5:** Association between awareness of 2011 consensus definition of cancer cachexia, ESPEN guidelines and other/local guidelines and screening for cancer cachexia (CC), clinical practice and clinician characteristics

Awareness of: screening^c^	2011 consensus definition of cancer cachexia (*n* = 79)		*p *value	ESPEN^a^ guidelines on nutrition in cancer patients (*n* = 79)		*p* value	Other guidelines including local guidelines (*n* = 79)		*p *value
	Yes (*n* = 33) *n* (%)	No (*n* = 46) *n* (%)		Yes (*n* = 17) *n* (%)	No (*n* = 62) *n* (%)		Yes (*n* = 16) *n* (%)	No (*n* = 63) *n* (%)	
Use a formal nutritional tool in screening for CC	8 (24.2)	9 (19.6)	0.78	7 (41.2)	10 (16.1)	0.04*	5 (31.3)	12 (19.0)	0.32
Screen for specific symptoms related to CC	28 (84.8)	31 (67.4)	0.12	14 (82.4)	45 (72.6)	0.54	14 (87.5)	45 (71.4)	0.33
Use pathology tests for screening for CC	20 (60.6)	14 (30.4)	0.01*	10 (58.8)	24 (38.7)	0.17	9 (56.3)	25 (39.7)	0.27
Use biometric data for screening for CC	12 (36.4)	14 (30.4)	0.63	9 (52.9)	17 (27.4)	0.08	6 (37.5)	20 (31.7)	0.77

Awareness of: clinical practice^b^	2011 consensus definition of cancer cachexia (*n* = 143)		*p *value	ESPEN^b^ guidelines on nutrition in cancer patients (*n* = 143)		*p *value	Other guidelines including local guidelines (*n* = 143)		*p *value
	Yes (*n* = 50) *n* (%)	No (*n* = 93) *n* (%)		Yes (*n* = 19) *n* (%)	No (*n* = 124) *n* (%)		Yes (*n* = 17) *n* (%)	No (*n* = 126) *n* (%)	
Is it part of your current practice to assess and treat cancer cachexia?	33 (66)	46 (49.5)	0.08	17 (89.5)	62 (50)	0.00*	16 (94.1)	63 (50)	0.00*

Awareness of: clinical practice^b^	2011 consensus definition of cancer cachexia (*n* = 59)		*p *value	ESPEN^b^ guidelines on nutrition in cancer patients (*n* = 59)		*p *value	Other guidelines including local guidelines (*n* = 59)		*p *value
	Yes (*n* = 28) *n* (%)	No (*n* = 31) *n* (%)		Yes (*n* = 14) *n* (%)	No (*n* = 45) *n* (%)		Yes (*n* = 14) *n* (%)	No (*n* = 45) *n* (%)	
Do you reassess these specific symptoms at each review?	21 (75)	23 (74.2)	1.00	12 (85.7)	32 (71.1)	0.48	10 (71.4)	34 (75.6)	0.74

Awareness of: clinician characteristics	2011 consensus definition of cancer cachexia (*n* = 176)		*p *value	ESPEN^b^ guidelines on nutrition in cancer patients (*n* = 165)		*p *value	Other guidelines including local guidelines (*n* = 166)		*p *value
	Yes (*n* = 62) *n* (%)	No (*n* = 114) *n* (%)		Yes (*n* = 24) *n* (%)	No (*n* = 141) *n* (%)		Yes (*n* = 22) *n* (%)	No (*n* = 144) *n* (%)	
Clinician by country^b^									
Australia	53 (85.5)	106 (93)	0.12	22 (91.7)	129 (91.5)	1.0	21 (95.5)	131 (91)	0.7
New Zealand	9 (14.5)	8 (7)		2 (8.3)	12 (8.5)		1 (4.5)	13 (9)	
Clinician by occupation^c^									
Medical doctor	31 (50)	48 (42.1)	0.05	11 (45.8)	65 (46.1)	0.00*	12 (54.5)	64 (44.4)	0.01*
Nursing	22 (35.5)	59 (51.8)		5 (20.8)	70 (49.6)		5 (22.7)	71 (49.3)	
Dietician/physiotherapist/occupational therapist/psychologist/social worker/others	9 (14.5)	7 (6.1)		8 (33.3)	6 (4.3)		5 (22.7)	9 (6.3)	

*p < 0.05 means there is an association between the variables

Reporting ‘yes’ responses

^a^*ESPEN* European Society for Clinical Nutrition and Metabolism

^b^Fisher’s exact test

^c^Pearson Chi-square test

Pearson chi-square test (Table 5) showed an association between occupation (medical doctors, nursing, dietitian/physiotherapist/occupational therapist/psychologist/social work/others) and ESPEN guidelines [*Χ*2 (2, *N* = 165) = 24.184, *p* = 0.00]; occupation and other/local guidelines [*Χ*2 (2, *N* = 166) = 9.514, *p* = 0.01] and borderline association with occupation and 2011 consensus definition [*Χ*2 (2, *N* = 176) = 5.967, *p* = 0.05].

## Discussion

This study demonstrates that the multifactorial nature of CC extends to the experiences of HCPs involved in its assessment and management. The survey revealed that HCPs assess their management of CC as poor. These issues were explored within three domains: knowledge, clinical practice and clinical management.

### Knowledge

Few HCPs were aware of key definitions or current guidelines. Consistent with previous studies, most respondents did not diagnose CC until weight loss ≥ 10% [18, 19], suggesting that early cachexia will be undiagnosed and untreated.

The lack of awareness of guidelines perhaps also reflects a recent review highlighting the heterogeneity within and between guidelines, illustrating the difficulty of advocating for particular guidelines [20]. Despite this, there was an association between assessing and treating CC and awareness of ESPEN/other guidelines and clinician occupation with 2011 consensus definition/ESPEN/other guidelines suggesting that HCPs are likely consulting guidelines for CC management, and thus the need for guidelines to be kept current. However, it should be noted that the awareness of guidelines is futile if clinicians are not reading, understanding or using the guidelines, and this needs to be advocated for at the workplace and/or in any training of CC.

In this study, nearly all respondents identified that formal training/education in CC would benefit their clinical practice to improve their confidence. These responses reflect previous studies which demonstrated that knowledge and understanding of CC and its effects on patients are essential for managing it [13, 15, 16, 21]. Nearly all respondents also agreed that further CC research and its impact on patients is required, reflecting the complexity of the syndrome. Most respondents were comfortable discussing the psychosocial impact of CC with their patients and carers, potentially reflecting the preponderance of respondents who worked in palliative care with its emphasis on the relief of physical and psychosocial suffering.

### Clinical practice

Recent studies have highlighted the significance of recognising cachexia as a debilitating syndrome [22] and the importance of specialised care for patients and their carers [15]. The multidisciplinary approach to CC is advocated to support the patient and carer, and can only work if each clinical discipline contributes [23]. With weight loss and anorexia being hallmarks of CC, it is concerning that only one fifth of respondents reported “regular review” by a dietitian despite current clinical guidelines [17, 24]. Most allied health involvement was ad hoc rather than a structured multidisciplinary approach to CC management contrasting with evidence that, for example, physiotherapists have a role to maintain and potentially rebuild muscle stores [25, 26]. Psychologists, social workers and counsellors were involved “as needed” which may suggest that they are not considered as core personnel in CC management and may be an area of under servicing in CC management [11, 27]. These results need to be considered in the light of a recent study demonstrating the positive effects of the multidisciplinary team in CC management [28]. It is noteworthy that the current American Society of Clinical Oncology (ASCO) guideline does not recommend any specific clinical disciplines [24].

Although there was a statistically significant association with formal nutritional screening tools and the ESPEN guidelines, very few respondents used them. The MST is mandatory within public hospitals in one state of Australia (NSW) and its use is supported by a study which showed that the tool had the greatest ability to detect cachexia among patients with gastric cancer when compared to other tools [29]. Around three-quarters of respondents screened for specific symptoms and reassessed these at each review, emphasising the importance of co-screening and symptom control [30, 31].

Biometric testing such as mid arm strength etc. was rarely used in clinical practice most likely indicating minimal understanding of its application. Weight or BMI is a simple diagnostic tool to indicate potential CC [32], but this was rarely recorded by clinicians. One study showed that 56% of hospice staff considered weighing would cause patient distress, while 96% of patients did not find weighing to be upsetting [33]. Missing this early prognostic sign could impact quality of life and the potential to address the syndrome [18, 34].

Diagnosing CC is complicated by poor agreement on biomarker criteria [35]. Pathology tests were rarely used by respondents. Interestingly, our results showed a statistically significant association between pathology screening for CC and awareness of the 2011 consensus definition which may indicate that these HCPs have knowledge of the diagnosis of CC.

The low use of assessment tools, such as quality of life, mirrored the low use of other screening tools which could reflect the lack of training in their application. Most respondents used “regular” symptom and “as needed” pathology assessments most likely reflecting standard practice of generic symptom and blood monitoring. A structured approach to assessment (e.g. combining PG-SGA and a symptoms and concerns checklist) and simple advice have been shown to reduce symptom burden, especially in people with advanced cancer [36]. Respondents were keen on a central repository for CC-related tools and information which may increase their uptake.

### Clinical management

Although over three-quarter of respondents indicated that CC management was an important part of their clinical practice, it was the carers who were more likely to initiate the discussion/management of CC, consistent with one previous study [37]. This may reflect HCPs’ reluctance to discuss cachexia with their patients because of the link to a poor prognosis [38].

Clinical management is made more difficult because there is no registered medication to manage CC (except in Japan), with most treatments geared toward appetite improvement [39]. Anorexia has a profound impact on a person’s oral intake, often worsening weight loss [40]. Responses reflected locally available therapies used for anorexia: antiemetics and prokinetics [17, 24].

There was an emphasis on nonpharmaceutical interventions—psychological support, nutrition counselling, exercise and mouth care which may reflect the lack of any definitive treatment. Nutritional intake may be significantly influenced by comorbidities and/or personal and cultural preferences, which adds additional complexity to advice, and further supports the inclusion of dietitians in managing CC. Notably, the ASCO guidelines do not recommend any interventions for managing CC [24].

The study’s strength is the inclusion of a range of health care disciplines and the holistic view of HCP’s management of CC, with domains and results comparable to studies undertaken in other countries. A limitation of this survey is the low response rate. This could indicate the HCPs’ current workload, unwillingness to complete a relatively long survey, or CC being a low priority in their practice.

## Conclusion

This study underlines the need for HCP training and national support for the systematic assessment and management of CC. Although access to a multidisciplinary team is ideal, a simplified, systematic approach incorporating screening, assessment and evidence-based interventions is recommended for ongoing patient care. Future research needs to examine whether earlier diagnosis of, and intervention(s) for, CC have positive impacts during cancer treatment and survival. It is recommended that the patient and carer experience of CC management be investigated to understand their perceptions and focus future research.

## Supplementary Information

Below is the link to the electronic supplementary material.

Supplementary file1 (DOCX 27 KB)

## Data Availability

The data that support the findings of this study are available from the corresponding author upon request.
