# Supplementary material for: Health care professionals’ experiences of dealing with cancer cachexia
Source: Int J Clin Oncol. 2023 Feb 23;28(4):592–602. doi: 10.1007/s10147-023-02300-6 (PMC10066081; doi:10.1007/s10147-023-02300-6)
Supplement: Supplementary file 1 — Supplementary file1 (DOCX 27 KB) [file 10147_2023_2300_MOESM1_ESM.docx]

**Health care professionals’ experiences of dealing with cancer cachexia**

**Journal: International Journal of Clinical Oncology**

Author names: Jodie Ellis, Michelle Petersen, Sungwon Chang, Gemma Ingham, Peter Martin, Nicola Morgan, Vanessa Vaughan, Linda Brown, David C. Currow, Valentina Razmovski-Naumovski

^*^Corresponding author

Dr Valentina Razmovski-Naumovski | BE (Hons), BSc (Chem.), PhD, GCULT, FHEA

Senior Research Fellow

South West Sydney Clinical Campuses - UNSW Medicine & Health

E: [v.naumovski@unsw.edu.au](mailto:v.naumovski@unsw.edu.au)

**Supplementary 1** Responses of other/open questions of the survey, including main themes (where applicable) and number (No.) of responses

| Question (Table) | Responses | No. |
| --- | --- | --- |
| **Table 1** **Demographics** |  |  |
| Current workplace – Other | Academic research/university | 2 |
|  | Aged care | 3 |
|  | Community | 2 |
|  | Day hospital | 2 |
|  | Disability | 1 |
|  | Rural hospital | 2 |
|  | Hospice | 2 |
|  | Oncology | 1 |
|  | Radiation | 3 |
|  | Strategy and governance | 1 |
|  | Support services | 1 |
| **Table 2 Knowledge** |  |  |
| Guidelines – Other | ASCO* (The American Society of Clinical Oncology) | 3 |
|  | ESMO* (The European Society for Medical Oncology) | 2 |
|  | BCCA* (BC Cancer Agency) | 1 |
|  | NCCN* (National Comprehensive Cancer Network) | 1 |
|  | DAA* (Dietitians Association of Australia Cancer Cachexia Guideline 2006). | 3 |
|  | CEDD* (Centre for Eating and Dieting Disorders) | 1 |
|  | COG* (Children's Oncology Group) | 1 |
|  | SIOP* (International Society of Paediatric Oncology) | 1 |
|  | CCLG* (Children's Cancer and Leukaemia Group) | 1 |
|  | Departmental/centre/local practice | 4 |
|  | COSA* (Clinical Oncology Society of Australia) Head and Neck Nutrition Guidelines | 1 |
|  | eTG complete - Palliative care | 1 |
|  | GCHHS* (Gold Coast Hospital and Health Service)/local service | 3 |
|  | Malnutrition scores/weight loss: refer to dietitians | 2 |
|  | Therapeutic guidelines | 2 |
|  | Palliative care formulary | 1 |
|  |  |  |
| Is further clinical research in CC and its impact on patients required – Themes | Problem (to patients/carers) | 30 |
|  | Need education/information | 17 |
|  | Need research | 17 |
|  | Need (evidence-based) treatment/interventions/management/tools/support | 41 |
|  | Priority/acknowledged/awareness (need for) | 12 |
|  | Unsolved; difficult to treat/reverse in (deteriorating) population | 10 |
|  |  |  |
| Is there anything that would increase your confidence level in managing advanced cancer patients with CC – Other | A belief that it is treatable | 1 |
|  | Ability of team | 1 |
|  | Effective treatments | 1 |
|  | Access to clinical studies | 1 |
|  | Dietitian support | 2 |
|  | (Only physio interventions- not able to provide symptom management) | 1 |
|  | Resources inc. multidisciplinary team | 2 |
|  | Know how it is affecting on the patient's life subjectively from patient's report | 1 |
|  |  |  |
| Training or education provider – Name | Minimal (as registrar)/lecture/specialist/advanced training | 5 |
|  | Postgraduate training | 2 |
|  | CC palliative care workshop/conference or seminar | 5 |
|  | PhD in CC | 2 |
|  | FAChPM* (Fellowship of the Australasian Chapter of Palliative Medicine)/FRACP* (Fellow of the Royal Australasian College of Physicians) | 1 |
|  | Journal paper(s)/guidelines | 3 |
|  | SIPM* (Sydney Institute of Palliative Medicine) /Sobell house | 2 |
| **Table 3 Clinical practice** |  |  |
| Which of the following personnel are involved – Other | Limited access to allied health (e.g. dietitian) | 1 |
|  | Exercise physiologist | 2 |
|  | Music therapists | 1 |
|  | Chaplain | 3 |
|  | Palliative Care | 1 |
|  | Pharmacist | 2 |
|  | Diversional therapist | 2 |
|  | Pastoral/Spiritual care | 10 |
|  | Social work | 1 |
|  | Ambulatory Care Nurses | 1 |
|  | Bereavement, community staff | 1 |
|  | Pathologist | 2 |
|  | Radiologist | 2 |
|  | PMA? | 1 |
|  | Librarian | 1 |
|  |  |  |
| Please indicate at what stage the following personnel are involved – Other |  |  |
| initial | Radiology, Consultant, Pathologist | 1 |
| ongoing | Pastoral care | 5 |
| ongoing | Chaplain | 2 |
| ongoing | Spiritual care | 2 |
| ongoing | Cultural care | 1 |
| ongoing | EP* (Exercise physiologist) | 2 |
| ongoing | Palliative care | 1 |
| ongoing | Palliative care nurse practitioner | 1 |
| ongoing | Pathology | 1 |
| ongoing | Radiology | 1 |
| ongoing | Pharmacist | 2 |
| ongoing | PMA? | 1 |
| ongoing | Volunteer | 1 |
| ongoing | Allied health | 1 |
|  |  |  |
| Are there any personnel that are not part of the multidisciplinary team due to lack of access/service provision issues – Other | EP* (Exercise physiologist) | 2 |
|  | Pastoral care | 1 |
|  | Grief/bereavement counsellor | 1 |
|  | Palliative care | 2 |
|  | Oncology | 1 |
|  | Pharmacist | 2 |
|  | Physiotherapy | 1 |
|  | Psychologist | 1 |
|  | Diversional therapist | 1 |
|  | Speech pathologist, dietitian, counsellor, occupational therapist (all in community setting) | 1 |
|  |  |  |
| Screening tools – Other | % Weight change | 1 |
|  | Fearon 2001 criteria | 1 |
|  | Review of dietary intake on admission and weight loss | 1 |
|  |  |  |
| Specific symptoms – Other | Availability of appropriate food | 1 |
|  | Taste change | 2 |
|  | Drowsiness | 1 |
|  | Pain | 2 |
|  | SOB* (Shortness of breath)/ Dyspnoea | 2 |
|  | Functional decline/strength | 1 |
|  | Inflammation CRP/albumin | 1 |
|  | Weight loss/Symptoms of weight loss | 2 |
|  |  |  |
| Pathology tests – Other | Glucose | 1 |
|  | Lymphopenia | 1 |
|  |  |  |
| Biometric test – Other | Bioelectrical impedance analysis/mBCA* (body composition analysis) | 4 |
|  | Sit to stand 30 | 1 |
|  | Utilise clinical rather than biometric due to pt* (patient) clear clinical dx (diagnosis) at time of referral | 1 |
|  |  |  |
| Assessments performed – initial only  – Other | Dietitian review | 1 |
|  | Ongoing support | 1 |
| Assessments performed – ongoing  – Other | Body composition | 1 |
|  | DT (Distress thermometer) and symptom checklist | 1 |
|  | Dynamometer grip, ESAS* (Edmonton Symptom Assessment System), TUG* (Timed Up and Go), Chair stand | 1 |
|  | Simple malnutrition screening tool at each presentation | 1 |
| Assessments performed – as needed – Other | PRN review | 2 |
|  | MST* (Malnutrition Screening Tool) | 1 |
| **Table 4** **Clinical management** |  |  |
| Pharmaceutical prescribed – Other | EPA/Fish oil | 6 |
|  | NSAIDS/analgesia/anti-inflammatory | 6 |
|  | Aperients | 1 |
|  | Cannot prescribe | 1 |
|  | Candidiasis treatment | 1 |
|  | Vitamins / (Zinc) | 1/ (3) |
|  | Dietitian review | 2 |
|  | Supplemental feeding or NGT feeds | 1 |
|  | Parenteral/enteral nutrition | 1 |
|  | Exercise | 1 |
|  | Ginger | 2 |
|  | (unable to prescribe cannabinoids) | 1 |
|  | Methylphenydate | 1 |
|  | Mirtazapine | 5 |
|  | Olanzapine | 1 |
|  | Antiemetics | 1 |
|  | Pain, nausea and symptom management | 1 |
|  | Proton pump inhibitors | 1 |
|  | Steroids | 1 |
|  | Watch cooking shows | 1 |
|  | Small meals | 1 |
|  | No | 5 |
|  |  |  |
| Nonpharmaceutical recommended – Other | CAM* (Complementary and alternative medicine) | 1 |
|  | Deprescribing | 2 |
|  | Fish oil | 3 |
|  | Family/general education | 2 |
|  | Outdoors | 1 |
|  | Relaxation | 1 |
|  | Yoga/tai chi | 2 |
|  | High Protein/nutritional supplement | 3 |
|  | Alcohol | 1 |
|  | Small amount of food more frequently | 1 |
|  | No | 3 |

CC = Cancer cachexia; *believed to be this acronym
